# Spatial analysis of measles cases and vaccination coverage in the Somali region, eastern Ethiopia

**DOI:** 10.3389/fepid.2025.1498750

**Published:** 2025-02-10

**Authors:** Abdilahi Ibrahim Muse, Mahdi Yonis Kayat, Mohamed Harir Aden, Jemal Beksisa Shuramu, Shikur Mohammed, Musse Ahmed Ibrahim, Binyam Mohammedbirhan Berhe, Ahmed Abdi Kalinle, Sahardiid Ali Abdilahi

**Affiliations:** ^1^ Regional Data Management Center for Health, Somali Region Health Bureau, Jigjiga, Ethiopia; ^2^ Public Health Department, Institute of Health Science, Jigjiga University, Jigjiga, Ethiopia; ^3^ National Data Management Center, Ethiopian Public Health Institute, Addis Ababa, Ethiopia; ^4^ Medicine Department, Institute of Health Science, Jigjiga University, Jigjiga, Ethiopia; ^5^ Health Bureau, Jigjiga, Somali Region, Ethiopia; ^6^ Public Health Emergency Directorate, Somali Region Health Bureau, Jigjiga, Ethiopia

**Keywords:** spatial analysis, measles incidence, vaccination coverage, Somali region, Ethiopia

## Abstract

**Background:**

Measles is a major public health concern that causes morbidity and mortality among children. In 2019, measles incidence reached its highest level in 23 years, with low measles containing vaccine dose one coverage playing a vital role. It can be prevented by two doses of the measles vaccine, either alone or in combination with measles-rubella (MR), which is a low-cost strategy for lowering morbidity and mortality among children.

**Objectives:**

To conduct spatial analysis of measles cases and vaccination coverage in the Somali region, Eastern Ethiopia.

**Methods:**

This retrospective study was done by using public health emergency directorate measles data from 2022 to 2023 and four years (July 2019–July 2023) of vaccination data from district health information system version 2.36. After the data completeness and consistency were ensured, it was cleaned and recoded. STATA version 17 and QGIS version 3.38 software were used for the data analysis.

**Results:**

From 2022 to 2023, the disease affected more than 5,930 people. The majority of the participants, 5,260 (88.7%), were under the age of 59 months, with 3,184 (53.7%) being male. Furthermore, the majority of residents were from Nogob 2,238 (37.7%), Erer 1,027 (17.3%), and Jarar 954 (16.1%). According to clinical symptoms, 5,930 (100%) of the cases had fever, cough, and rash, and more than two-thirds, 4,901 (82.6%), had complications. A measles vaccination coverage of 59.4% and a measles incidence of 0.087 per 100 people were found in the region.

**Conclusions:**

This study found a very low measles vaccination coverage. Furthermore, Nogob, Erer, and Jarar zones showed the highest measles incidence rate, respectively. It is recommended to strengthen routine immunization services according to the national vaccination agenda, categorize, and reach unvaccinated children through catch-up vaccination campaigns. A concerted effort should be made to improve MCV2 coverage in hard-to-reach areas of the region. Special focus should be given to vaccine cold chain management in the zone and its districts with high vaccination coverage but also a high measles incidence rate. An investigation should be done into the associated factors of the higher incidence despite its vaccination coverage.

## Introduction

Measles, a vaccine-preventable disease, is a major public health concern that causes morbidity and mortality among children (1, 2). It is a very infectious disease and one of the most transmittable illnesses known to people, caused by the measles virus, an RNA virus in the Morbillivirus genus (3, 4). Contact with contaminated nasal or throat secretions, as well as breathing air that has been inhaled by someone who has measles, spreads the disease. For up to two hours, the virus remains active and contagious in the air or on infected surfaces (3). After exposure or contact with respiratory droplets, 90% of susceptible individuals can develop systemic infection within 10–14 days. The prodromal phase is characterized by fever, malaise, and the C's (cough, coryza, and conjunctivitis), with Koplik's spot and an erythematous maculopapular rash being two common findings at this stage (5).

Acute measles infection at an early age is associated with more complications and increased mortality; the most prominent consequences of measles causing morbidity and mortality are bronchopneumonia, otitis media, diarrhea, croup, and encephalitis (6). Vitamin A is given to all measles cases for treatment and all cases are handled symptomatically with supportive measures (7). Vaccination is a low-cost strategy to reduce morbidity and mortality among children (8). Measles can be prevented by two doses of measles vaccination, either alone or in combination with measles-rubella (MR), measles-mumps-rubella (MMR), or measles-mumps-rubella-varicella (MMRV) (9). Measles-containing vaccine dose one (MCV1) is recommended at 9 months and the second dose (MCV2) at 15–18 months (10). These are programmatically scheduled vaccines that can be provided through regular services, intensified routine services, or supplementary immunization activities (11).

Measles vaccination coverage is the proportion of people who have received measles vaccine doses one and two. Due to COVID-19 effects, millions of youngsters have missed measles immunizations, resulting in epidemics all over the world (12). In 2019, measles incidence reached its peak level in 23 years, primarily due to large outbreaks in multiple countries, with low MCV1 coverage being a significant factor (13). Measles continues to be an important public health problem (1). In 2022, 136,000 measles deaths occurred globally, primarily among unvaccinated children under 5 years old. 83% of children received one dose of vaccine by their first birthday, the lowest since 2008 (3).

Measles is highly contagious, so its outbreaks are crucial indicators of inadequate vaccine coverage and deficiencies in the health system. From 2006 to 2016, there was a gradual decline in the vaccine coverage (VC) of the measles-mumps-rubella (MMR) in Brazil, with a yearly reduction of 2.7% (14). From 2005 to 2011, China's MCV vaccination coverage for children was 98.6% for MCV1 and 82.8% for MCV2, much below the threshold for maintaining herd immunity (15). Furthermore, measles vaccine coverage in Pakistan fell between 2012 and 2013, resulting in 30,000 measles cases being reported in the country (16). There have also been a lot of measles cases in Myanmar in recent years, which have been linked to inadequate vaccination rates (17). Similarly, Bangladesh has recorded measles vaccine coverage below the intended level, leading to significantly higher rates of morbidity and mortality in the nation (18).

In underdeveloped countries, measles-related deaths account for roughly 10% of all deaths in children under the age of 5 years. It is also responsible for 6%–21% of all instances of acute lower respiratory infections and 8%–50% of all pneumonia-related deaths (6). According to Africa, there are numerous challenges that could affect the use of vaccination services, In Gambia, it was reported that the country's national MCV1 coverage ranged between 90% and 97% from 2011 to 218 and fell to 85% in 2019 (19). Including the Democratic Republic of Congo, 2019 marked the highest measles-related deaths in two decades, with endemic transmission re-establishing in countries with previously achieved elimination targets. According to the Horn of Africa, the measles vaccination coverage is 56.2%, 64%, 85%, and 59% in Kenya, Sudan, Eritrea, and Ethiopia, respectively (1, 9, 20, 21). Despite routine immunizations and supplemental immunization activities in the country as a whole and in the Somali region in particular, measles outbreaks occur on a regular basis. The Somali region has the highest reported rates, with 540 cases per one million inhabitants (22). The findings of this study will be beneficial to all under-five children who live in the Somali region, the regional health bureau, and its partners who work in the area of health. Moreover, this study will be a base for future researchers.

Detecting inhabitants with low measles vaccination coverage can guide supplemental immunization efforts and enhance elimination programs (9). So far, no study has investigated the spatial analysis of measles cases and the trends of measles vaccination coverage at the study area. Therefore, this study aimed to explore the regional geographic distribution of measles cases and trends in measles vaccine coverage.

## Methods and materials

### Study setting and period

**Figure 1:**
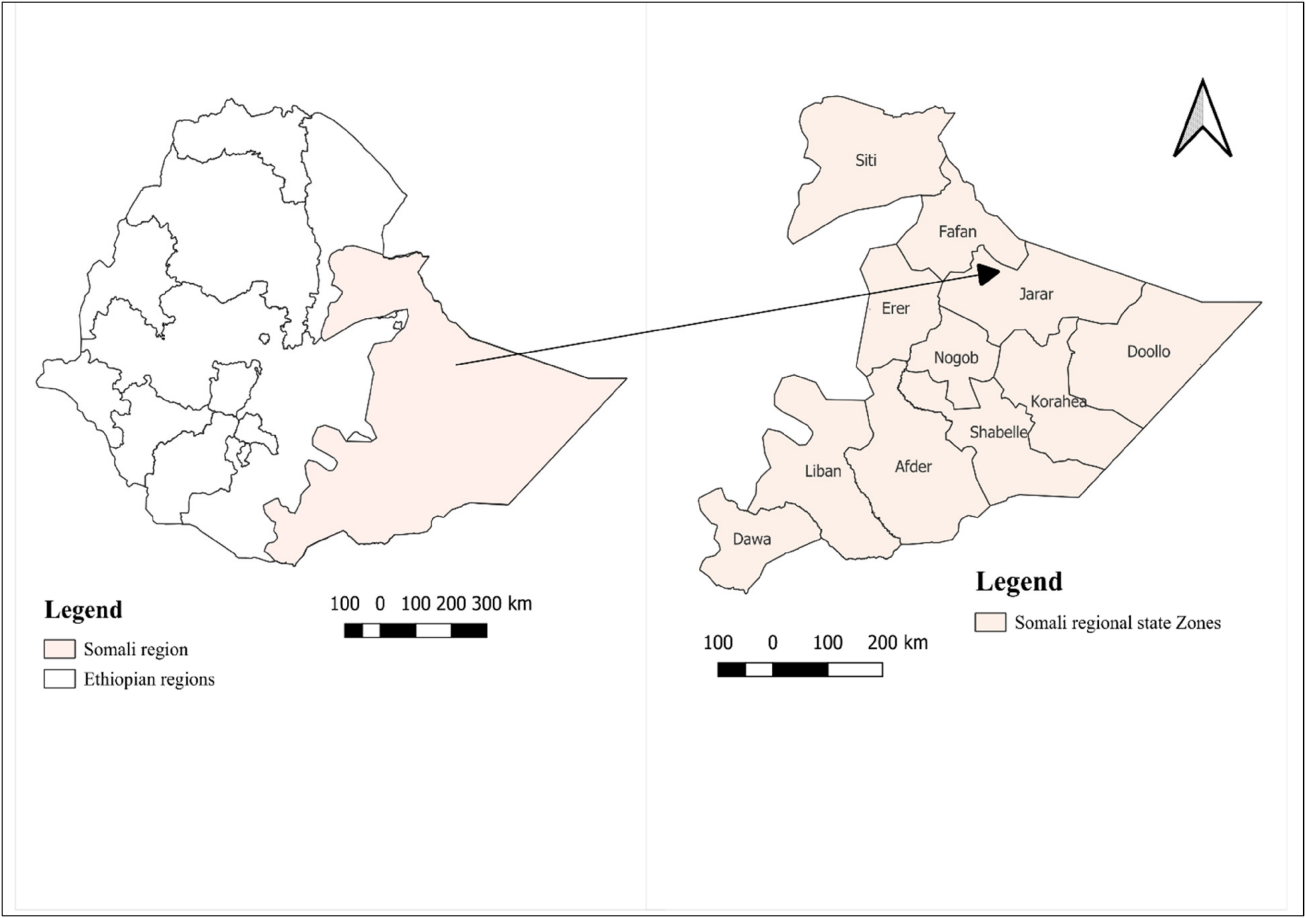
Study area map.

Ethiopia has 12 regions and 2 administrative cities, one of which is the Somali region. The region is located in the eastern part of Ethiopia and covers an area of over 375,000 km^2^. Its temperatures range from 18 to 45 degrees Celsius, and its annual rainfall is from 386 to 660 mm. The Somali region has a population of 6.8 million people, of which 86% live in rural areas and 14% in urban areas (23). Furthermore, there are 1,596 health posts, 227 health centers, and 18 hospitals in terms of health institutions. The study period was from May 1st, 2024, to June 30th, 2024 (Figure 1).

### Study design

A retrospective study was carried out in the Somali region using PHEM and DHIS 2 data.

### Population

All populations living in the Somali region were the source population, while patients confirmed with measles by polymerase chain reaction (PCR) and those subsequently confirmed by clinically or epidemiologically linked with measles cases were the study population.

### Eligibility

This study included patients who had been diagnosed with measles and had completely filled line lists, as well as measles vaccine coverage of children aged 9–12 months and 15–18 months from the Somali region's district health information system two (DHIS2).

### Data source

Secondary data (measles line list) from the Somali region's public health emergency directorate and district health information system version 2.36 (DHIS2) from the region was used to study the spatial analysis of measles cases and vaccination coverage in the Somali region. The investigators wrote a request letter to the public health emergency directorate requesting the aforementioned data and received it from the directorate. Furthermore, laboratory results from human samples collected during the outbreak period were used to summarize the outbreak.

### Study variables

The variables studied were patient characteristics, zones, districts, laboratory samples taken, clinical signs and symptoms, complications, treatment outcome, vaccination coverage, and incidence.

### Data quality checks

Since the data was in a line list, accuracy, consistency, completeness, and validity were all checked.

### Operational definitions

A clinically confirmed case is the one that has fever, cough, and coryza.

Laboratory-confirmed measles case is a case that meets the clinical case definition and has been confirmed by polymerase chain reaction (PCR) of measles virus infection (24).

**EPI-linked** is a clinical case of measles that was not proven by a laboratory but was geographically and chronologically related, with rash onset dates 7–21 days apart from a laboratory-confirmed case or another epidemiologically linked case (25).

**Spatial analysis** is the study of entities through the examination, assessment, evaluation, and modeling of spatial data characteristics such as locations, attributes, and relationships that reveal geometric or geographic properties.

### Data processing and analysis

The collected data were checked for completeness and consistency. The data were cleaned, recoded and exported to STATA version 17 software for analysis. Descriptive statistics was computed using frequency and percentages. Furthermore, QGIS version 3.38 was used for the spatial analysis of the measles vaccination and its incidence rate in the region.

## Results

### Sociodemographic characteristics of the study participants

This study covered measles cases from 2022 to 2023 and vaccination coverage from 2019 to 2023. In these two years, the disease affected more than 5,930 people. According to the characteristics of the participants, the majority of them, 5,260 (88.7%), were aged less than or equal to 59 months, and more than half of these, 3,184 (53.7%), were male. Concerning the zonal residence, the majority came from Nogob 2,238 (37.7%), Erer 1,027 (17.3%), and Jarar 954 (16.1%); and the majority came from Garbo 1,296 (21.9%), Jigjiga 728 (12.3%), and Degahbour 442 (7.5%), according to their district (Table 1).

**Table 1:** Sociodemographic characteristics of the participants, Somali region, eastern Ethiopia.

Variable	Category	Number	Percent
Age	≤59 months	5,260	88.7
	>59 months	670	11.3
Sex	Male	3,184	53.7
	Female	2,746	46.3
Zones	Afder	53	.9
	Dollo	296	5.0
	Erer	1,027	17.3
	Fafan	942	15.9
	Jarar	954	16.1
	Korahey	169	2.8
	Liban	12	.2
	Nogob	2,238	37.7
	Shabelle	95	1.6
	Siti	144	2.4
Districts	Garbo	1,296	21.9
	Jigjiga	728	12.3
	Degahbour	442	7.5

### Health facility and laboratory results report of the study participants

Health service institutions in the region reported all the cases. A hundred twenty seven (2.1%) of the participants were taken from a sample, of whom 98 (77.2%) were confirmed. More than half of the participants, 3,222 (54.2%), were admitted, while 120 deaths (2.0%) were documented (Table 2).

**Table 2:** Health facility and laboratory results report of the participants in the Somali region, eastern Ethiopia.

Variable	Category	Number	Percent
Health facility	Hospitals	2,066	34.8
	Health centers	2,725	46.0
	Health posts	1,139	19.2
Specimen taken	Yes	127	2.1
	No	5,803	97.9
Laboratory result (*N* = 127)	Confirmed	98	77.2
	Negative	29	22.8
Admission status	Inpatients	3,222	54.3
	Outpatients	2,708	45.7
Outcome	Alive	5,810	98
	Died	120	2

### Clinical signs and complications of the participants

According to the clinical signs of the measles cases in the Somali region, all 5,930 (100%) of the participants experienced fever, cough, and rash, However, 237 (4%) and 20 (0.3%) did not have coryza and conjunctivitis, respectively. Furthermore, more than two-thirds of the participants, 4,901 (82.6%) presented medical complications (Table 3).

**Table 3:** Clinical signs and complications of measles cases in the Somali region, eastern Ethiopia.

Variable	Category	Number	Percent
Fever	Yes	5,930	100
	No	0	0
Rash	Yes	5,930	100
	No	0	0
Cough	Yes	5,930	100
	No	0	0
Running nose coryza	Yes	5,693	96
	No	237	4
Red eyes conjunctivitis	Yes	5,910	99.7
	No	20	0.3
Complications	Yes	4,901	82.6
	No	1,029	17.4
Length of stay at home	0–1 day	841	14.2
	2–4 days	3,615	61.0
	≥5days	1,474	24.9

### Trends of measles vaccination coverage in the Somali region, eastern Ethiopia

**Figure 2:**
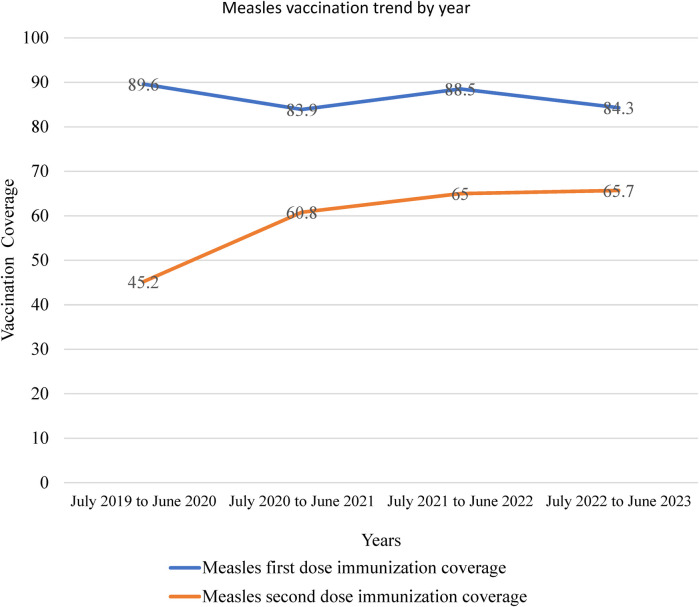
Trends of measles vaccination coverage in the Somali region, eastern Ethiopia.

The measles vaccination coverage was analyzed from July 2019 to June 2023. The coverage for the first dose of the measles-containing vaccine decreased from 89.6% to 84.3% while the coverage for the second dose ranged from 45.2% to 65.7% in the region (Figure 2).

### Somali region measles vaccination coverage of the study participants by zone

**Figure 3:**
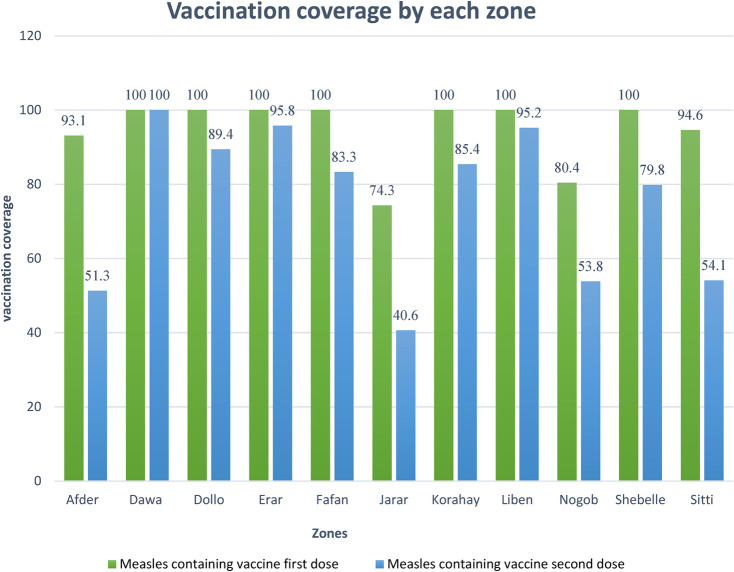
Somali region zonal measles vaccination coverage of the study participants.

Based on the zones of the Somali region and their immunization coverage for the last four years, the minimum coverage of measles containing vaccination dose one was Jarar at 74.3% and Nogob at 80.4%, while that of measles containing vaccine dose two was Jarar at 40.6% and Afdere at 51.3% (Figure 3).

### Measles vaccination coverage and incidence in Somali region

**Figure 4:**
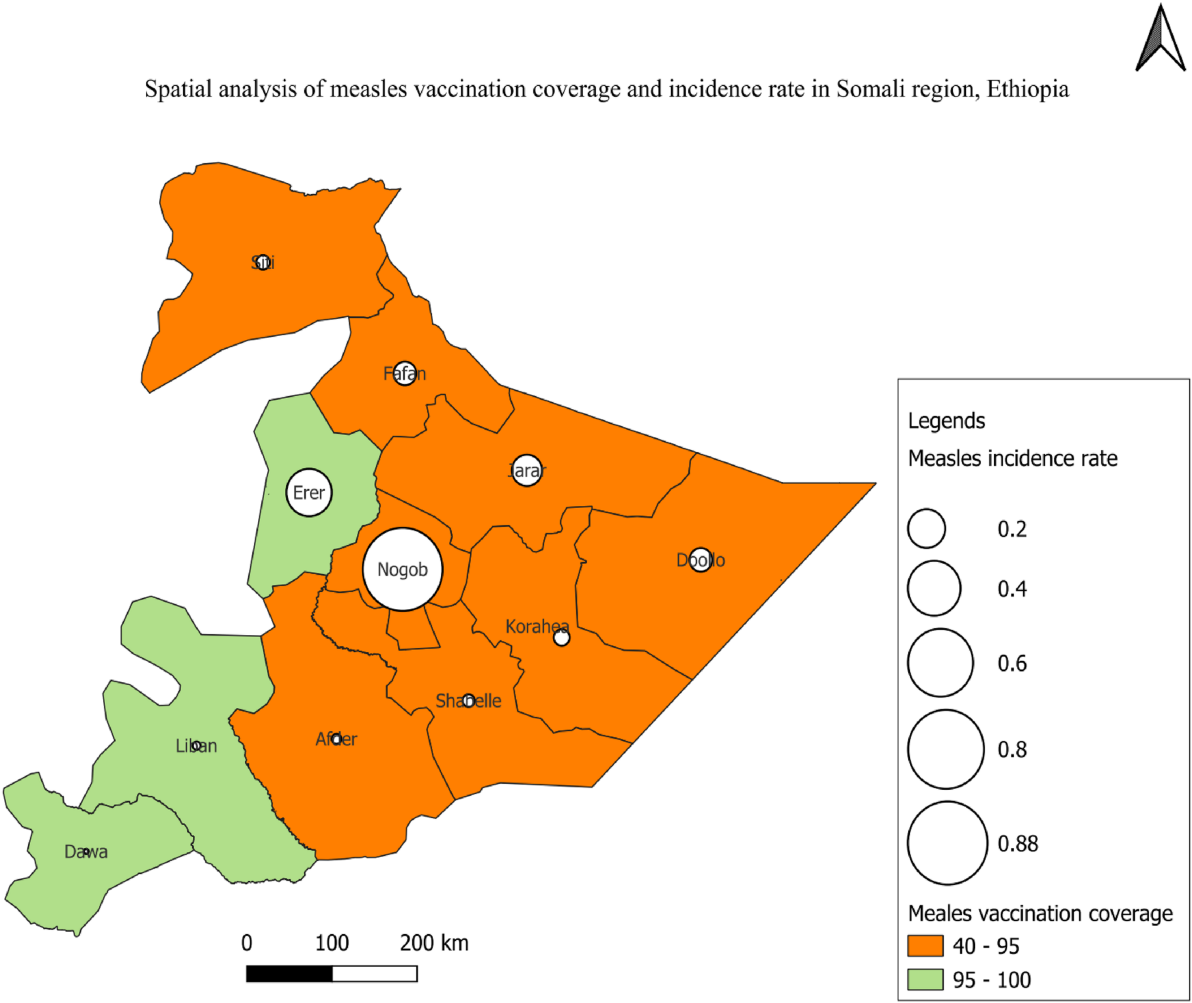
Vaccination coverage and the incidence of measles cases in Somali region, eastern Ethiopia.

Jarar (40.6%), Nogob (53.8%), Sitti (54.1%), and Afder (51.3%) had the lowest measles vaccination coverage among the zones of the Somali region, and the measles incidence rate was the highest in Nogob, Errer, and Jarar zones (Figure 4).

## Discussion

This study reported that more than half (54.3%) of the cases were admitted; that might be because of the region's poorly developed road infrastructure and, at the same time, the very widespread health service institutions. Furthermore, more than 80% of the population are pastoralists that frequently move where their herds can get pasture. These issues might have made the patients come to the health facilities when in critical conditions or with complications that might make the admission rate more than half. In addition, it was also discovered that the majority of the participants had medical complications, which might be because of their prolonged home stay with the disease.

Based on the zonal measles vaccine coverage and incidence rate, it was revealed that Erar zone has the second highest incidence rate, despite its high vaccination coverage. This might be because Erar is one of the region's most hard to reach zones. As a result, the high vaccination coverage and high incident rate might be attributed to either a poorly maintained cold chain system, such as sun exposure and inappropriate vaccine storage in the fridge, or the reporting system.

The result of this study showed that from 2019 to 2023, the measles-containing vaccine first dose decreased from 89.6% to 84.3%, which might be due to the COVID-19 effects of the policy priority shift. Furthermore, the measles-containing vaccine second dose was also slowly progressive from 45.2% to 65.7%, which means in a period of four years, only 20.5% progress was made and yearly 5%, which is very low. In comparison, the vaccination coverage of measles containing vaccine dose one was higher than the measles containing vaccination dose two coverage in each year, indicating that there are many children who drop out of the vaccination and are not fully immunized to protect against measles virus infection. The World Health Organization's goal is to eradicate measles by the end of 2030. To attain this target, countries need to obtain more than 95% coverage for both the first and second doses of the measles vaccine to create herd immunity (26). However, coverage in the Somali region is lower than expected.

This study found a low measles vaccination coverage (MCV2) of 59.4% (95% CI: 57.75, 60.25) and an incidence rate of 0.087 per 100 persons. This vaccination coverage is lower than studies done in China, which are 95.8% (27) and 93.9% (15). Moreover, this study's coverage is also lower than other studies carried out in Bangladesh and Bangalore, which showed 89.23% (28) and 83.3% (29), respectively. The reason for this difference might be that this study was implemented in a province of Ethiopia where the health service statistics are very low and most of the people are pastoralists who are more scatted and are less accessed to health service institutions compared to those studies conducted in China, Bangladesh, and Bangalore, where people are in cities and more aware of the vaccine.

Based on reports from Africa, the finding of this exploration is higher than a pooled prevalence of sub-Saharan Africa (44.77%) (10) and those carried out in Ghana (18.2%) (30) and Kenya (51.1%) (31). The reason that this report's finding is higher could be the differences in the study's methodology. In this study, the data was obtained from DHIS 2, where the data is filled directly from the child's report after vaccination, which makes the data actual, while the other studies used checklists and interviewed the caregivers or parents about the child's immunization status, which depends on the knowledge and recall memory of the caregivers.

In Ethiopia, the result of this study is lower than those that have been done in Assosa (71.77%) (32), Debreburhan (73%) (33), Debre Markos (91.7%) (34) and Bassona Worena (71.3%) (35), but in lined with a study done in Northwest Ethiopia (58.4%) (36). The reason for the difference could be differences in the methodology used, the data source, and the study area. These studies were carried out in urban settings, while this study was conducted region-wise. The region is one of the least developed among the states of Ethiopia, and frequent droughts hit it, which makes people frequently move from one area to another and makes them in a hard-to-reach location.

### Limitations of the study

The limits of the study are the nature of the study design and the secondary data studied.

## Conclusion and recommendations

The main findings of this study are a low measles vaccination coverage in which Jarar, Nogob, Sitti, and Afder zones had the lowest measles vaccination coverage. According to the participants’ clinical signs and symptoms, they all experienced fever, cough, and rash, and the majority had medical complications.

It is recommended to reinforce routine immunization services to confirm that all children obtain both doses of the measles-containing vaccine according to the national vaccination agenda, categorize and reach unvaccinated children, such as through catch-up vaccination campaigns, and progress public engagement and inoculation demand. A concerted effort should be made to improve MCV2 coverage in hard-to-reach areas of the region. Mothers should be encouraged to exclusively breastfeed their children during antenatal and postnatal care and to bring their children to health institutions as soon as possible when they get sick to reduce home stay duration. The area that has been found to have high vaccination coverage, which is in line with WHO recommendations, but had the second highest measles incidence rate. The vaccine cold chain management of that area should be ensured, and factors associated with the higher incidence despite its coverage should be investigated.

## Data Availability

The raw data supporting the conclusions of this article will be made available by the authors, without undue reservation.
